# Overexpression of *Sir2* in the adult fat body is sufficient to extend lifespan of male and female *Drosophila*

**DOI:** 10.18632/aging.100553

**Published:** 2013-04-29

**Authors:** Julia Hoffmann, Renja Romey, Christine Fink, Li Yong, Thomas Roeder

**Affiliations:** ^1^ University of Kiel, Dept. of Zoophysiology II, 24098 Kiel, Germany; ^2^ Biocenter Grindel and Zoological Museum, AG Molekulare Evolutionsbiologie, 20146 Hamburg, Germany

**Keywords:** Sir2, lifespan, fat body, dietary restriction, fat metabolism, lipid droplet, gene expression

## Abstract

*Sir2* is the most intensively discussed longevity gene in current aging research. Although, the gene encoding for a NAD^+^-dependent histone deacetylase initially was found to extend lifespan of various organisms ranging from yeast to mammals, serious doubts regarding its role in longevity have been expressed recently. In this study, we tested whether tissue-specific overexpression of *Sir2* in the adult fat body can extend lifespan when compared to genetically identical controls. We also wanted to elucidate the mechanisms by which fat body *Sir2* promotes longevity by studying the phenotypic and transcriptional changes in the fat body. We found that moderate (3-fold) *Sir2* overexpression in the fat body during adulthood only can promote longevity in both sexes by roughly 13 %. In addition, we obtained transcriptional profiles elicited by this overexpression and propose a role for *Sir2* in lipid droplet biology especially under conditions of starvation. Furthermore, our data do not support the idea of *Sir2* mediating the response to dietary restriction (DR) because transcriptional profiles of fat bodies after DR or *Sir2* overexpression do not match. This study provides additional independent evidence for the concept of *Sir2* as a longevity gene and as a promising pharmacological target to cure age-related diseases.

## INTRODUCTION

In current aging research, *Sir2* is one of the most prominent longevity genes and at the same time the most intensively discussed one. Kaeberlein et al. described *Sir2* (silent information regulator 2) as a NAD^+^-dependent histone deacetylase that extends replicative lifespan of yeast [1]. It is highly conserved in eukaryotes and lent its name to the family of Sirtuins (*Sir2-* like genes), which has five members in *Drosophila* (*Sir2*, *Sirt2, Sirt4, Sirt6* and *Sirt7*) and seven members in mammals.

Findings in *C. elegans* and *Drosophila* confirmed a role of *Sir2* on lifespan. Tissenbaum and colleagues showed that an increased copy number of *Sir2.1* extends lifespan of worms by up to 50 % [2]. In *Drosophila*, ubiquitous, constitutive overexpression as well as panneuronal overexpression only in the adult animal was shown to extend lifespan [3, 4]. Studies in mice have demonstrated that the *Sir2* homolog S*irt1* does not increase life expectancy but promotes a longer health span by prevention of age associated diseases [5-7].

Due to the apparent evolutionarily conserved effect of *Sir2*/S*irt1* on health and ageing, the physiological functions and protein targets of the enzyme have been intensively studied in mammals in order to cure age-related diseases. SIRT1 was shown to increase insulin sensitivity and regulate energy expenditure through stimulation of oxidative metabolism like fatty acid oxidation and oxidative phosphorylation in mice [5, 8-10]. This predestines the enzyme as a novel target for the treatment of diabetes [11]. In addition, it has been implicated in the etiology and/or cure of cancer, Alzheimer's and other age-related diseases [12-14]. In contrast to mammals, in *Drosophila* only few studies tried to elucidate physiological functions of *Sir2* and the molecular mechanisms, which may mediate its effect on life span. A screen for obesity-inducing genes in *Drosophila* larvae pointed to a role for *Sir2* in regulating fat metabolism and response to amino-acid starvation [15]. Moreover, *Sir2* apparently regulates expression of genes involved in fat metabolism and the lack of *Sir2* increases fat deposition under normal conditions and consequently impairs starvation survival of flies [16].

It has been hypothesized that *Sir2* mediates the effects of dietary restriction (DR) on fly lifespan because decreased activity of the histone deacetylase RPD3, which is down-regulated in DR, increases SIR2 activity [3]. This hypothesis is corroborated by the recent results that revealed a diet-dependent effect of *Sir2* on lifespan of *Drosophila* [17]. In addition, P53 is discussed as a downstream target of RPD3 and SIR*2* in mediating longevity [4].

Strong doubts over the lifespan prolonging effect of *Sir2* overexpression have recently been expressed [18]. As different genetic backgrounds of control and experimental animals can be a confounder in lifespan experiments, Burnett et al. [19] aimed to validate results for *C. elegans* and *Drosophila* with genetically matched controls and could not confirm the positive effect on lifespan. However, a very recent study has supported the initial observation by showing that genetic manipulation of *Sir2* in the fly's fat body elongates lifespan in *Drosophila* [17]. Thus, an urgent need to either confirm or rebut these effects of *Sir2* expression levels on lifespan exists. We used the inducible GeneSwitch Gal4/UAS system [20] to drive *Sir2* overexpression in the fat body only, which offers two major advantages: first, it is possible to restrict overexpression to the adult animals without affecting development and second, *Sir2* overexpressing flies and control flies have the same genetic background since overexpression is induced by treatment with mifepristone (RU486). We found that *Sir2* overexpression in the fat body only can promote longevity in both sexes. Furthermore, a detailed analysis of the fat tissue revealed no overt structural changes. However transcriptional profiles of fat bodies after DR or *Sir2* overexpression suggest that the interventions promote longevity through independent mechanisms.

## RESULTS

### Moderate Overexpression of *Sir2* in the Fat Body Elongates Lifespan of *Drosophila*

To avoid differences in genotypes between control and experimental animals, we used the inducible GeneSwitch System [20] to overexpress *Sir2* in the fat body of adult flies. Here, tissue-specific overexpression of *Sir2* is achieved by administration of mifepristone (RU486) to the F1 generation of a cross of UAS-*Sir2* with the driver line S_1_106-Gal4 while genetically and developmentally identical controls feed on normal medium supplemented with the same dose of the diluent ethanol. We first examined the expression level of *Sir2* under the experimental conditions. UAS-*Sir2*>S_1_106-Gal4 flies were fed 400 μM RU486 or diluent only for 24 h. Fat bodies were dissected and qPCR for *Sir2* mRNA was performed. We detected an approx. 3.5 fold and 2.5 fold up-regulation of *Sir2* gene expression in the fat bodies of females and males, respectively (Fig. S1). This system induces a moderate increase of *Sir2* expression, which is presumably in the physiological range of expression changes.

**Figure 1:**
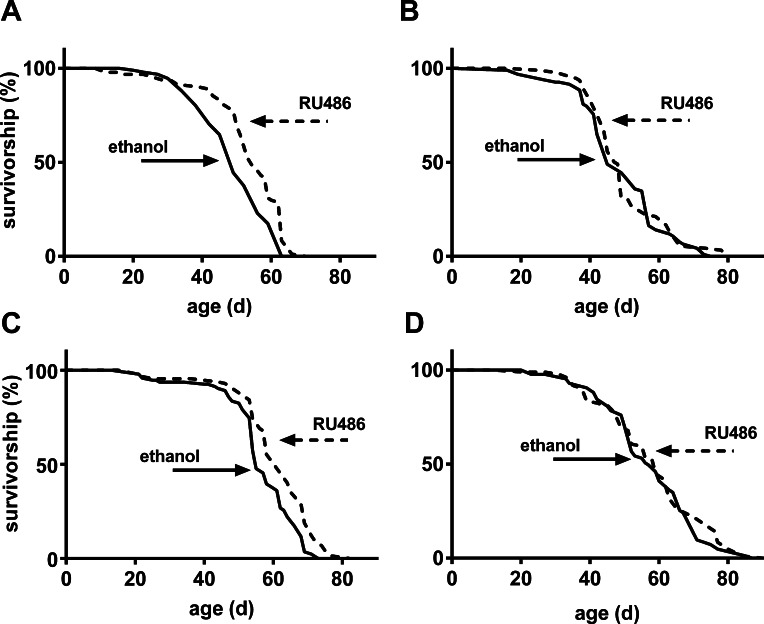
Increased expression of *Sir2* in the fat body extended lifespan of male and female flies Mated female (**A**) or male (**C**) UAS-*Sir2*>S_1_106-Gal4 flies were fed 400 μM RU486 or diluent (ethanol) throughout adult life and lifespan was determined. As a control for a potential lifespan extending effect of RU486, the driver line was crossed to *w^1118^* and female (**B**) or male control flies (**D**) of the F1 generation were treated as the experimental flies. For p-values of log rank test see Table 1, n = 100 for each group.

Until recently, studies that aimed to investigate the effect of *Sir2* on lifespan mainly manipulated its gene expression in whole animals or the nervous system. We decided to study the effect of overexpressing *Sir2* in the fat body as it is strongly expressed in this tissue [3]. Moreover, the fat body integrates the response to both immune and metabolic stimuli, which are both important modulators of the aging process [21, 22]. To do so, we determined lifespan of mated UAS-*Sir2*>S_1_106-Gal4 flies fed on medium with or without RU486 throughout adult life. Survival curves of females (Fig. 1A) and males (Fig. 1C) overexpressing *Sir2* are significantly shifted to the right. As shown in Table 1, median lifespan of females and males treated with RU486 is increased by 12 % and 13 % compared to controls, respectively. These data show that even the relatively weak overexpression of *Sir2* induced by the S_1_106-Gal4 line in the adult fat body promotes longevity in flies, largely by delaying the onset of cohort aging. To rule out the possibility that the GAL4 activator RU486 itself may elongate lifespan of *Drosophila*, we performed lifespan experiments with flies from a crossing of the *w^1118^* strain and the driver line S_1_106-Gal4. Flies were treated as described above and no significant shift in survival curves was observed for either females (Fig. 1B) or males (Fig. 1D) compared to controls.

**Table 1:** Elevated expression levels of *Sir2* increased life expectancy of flies

Genotype	Sex	RU486	Median lifespan (d)	Median lifespan extension (% of control)	Maximum lifespan (d)	χ2	p-value
UAS-*Sir2>*S_1_106-Gal4	♀	−	49	12.3	59	8.796	0.003
UAS-*Sir2>*S_1_106-Gal4	♀	+	55		62		
UAS-*Sir2>*S_1_106-Gal4	♂	−	55	12.7	68	13.15	0.0003
UAS-*Sir2>*S_1_106-Gal4	♂	+	62		71		
w^1118^*>*S_1_106-Gal4	♀	−	45	6.7	63	0.352	ns
w^1118^*>*S_1_106-Gal4	♀	+	48		63		
w^1118^*>*S_1_106-Gal4	♂	−	57	3.4	70	0.015	ns
w^1118^*>*S_1_106-Gal4	♂	+	59		76		

Data derived from log rank analysis of the survival curves shown in Fig. 1. The maximum lifespan is the median of 10 % survival. Log rank test was performed using GraphPad Prism 6.00. ns, not significant. Experiments shown are representatives of two independent experiments.

### Gene Expression Profiles Induced by *Sir2* Overexpression and Dietary Restriction Do not Match

**Figure 2:**
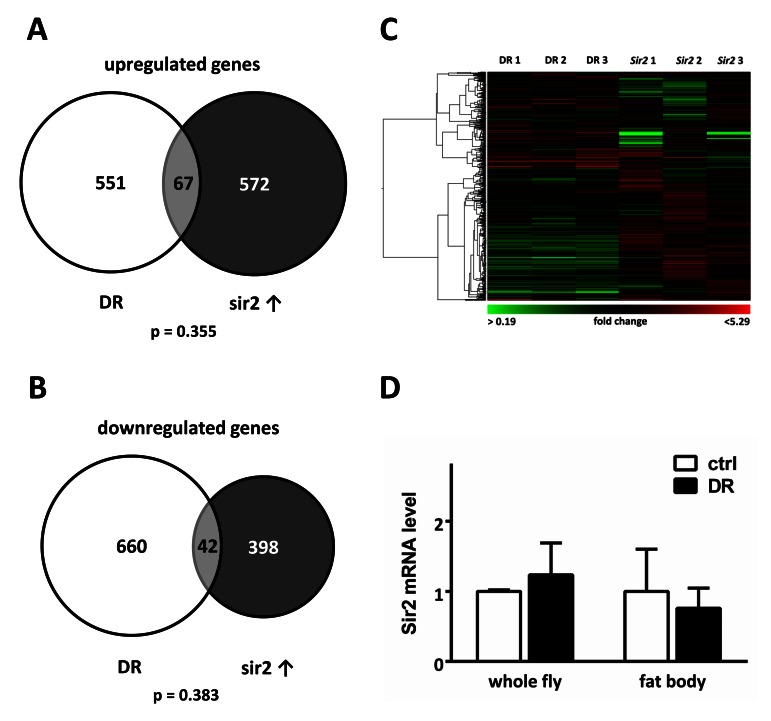
Fat body *Sir2* does not play a major role in the DR response Transcriptional profiles of fat bodies from either w1118 females after a 7 d DR period or UAS-*Sir2*>S_1_106-Gal4 females after 24 h of *Sir2* overexpression were compared. The scaled Venn diagrams depict the actual relationship of the numbers of up-regulated (**A**) and down-regulated genes (**B**) of both samples and the number of overlapping genes. Only roughly 10 % of differentially expressed genes are overlapping between DR samples and *Sir2* overexpression. Statistical analyses were performed with Fisher's exact test. Genes, which were up-or downregulated > 1.5 fold in at least 2 of 3 microarrays were included in the diagrams shown. Hierarchical clustering of DR and *Sir2* Arrays reveals that transcriptional patterns elicited by DR and *Sir2* overexpression in the fat body do not show substantial similarities. (**D**) mRNA levels of *Sir2* in female *w^1118^* whole animals and in their fat bodies were unchanged after DR. Shown are mean values of 3 replicates + SEM.

The finding that fat body *Sir2* can promote a long life led us to examine which candidate mechanisms may play a role in conferring *Sir2*-dependent longevity. As several studies suggest that *Sir2* mediates at least parts of the response to dietary restriction (DR)[3, 17] we aimed to elucidate how these two interventions alter gene expression patterns of the fat body and to what extent transcriptional responses match. We performed microarray analysis with RNA isolated from fat bodies of mated, age-matched females either subjected to DR for 7 d or with fat body specific *Sir2* overexpression after 24 h induction by RU486. The control and DR food was prepared according to recommendations by Ja et al. [23] to prevent desiccation, which robustly resulted in prolonged lifespan of male and female *w*^1118^ flies (Fig. S2). Surprisingly, when comparing gene expression profiles we noticed that only roughly 10 % of up- (p value 0.355) or down-regulated genes (p value 0.383) overlap between both samples (Fig 2A, B). This is a surprising small overlap, indicative for almost completely independent processes, especially when taking into account that fat tissue of age- and gender-matched flies was used for both analyses.

Furthermore, hierarchical clustering of the microarray data shows substantial differences in gene expression patterns induced by both interventions (Fig 2C). Especially genes down-regulated in DR sets are up-regulated in *Sir2* gene sets. Additionally, we did not detect an increase of *Sir2* transcription in response to DR as reported by other groups, neither in whole animals nor in their fat tissues (Fig. 2D). Taken together, these results imply that fat body *Sir2* evokes other cellular processes than those elicited by DR.

### Fat Body *Sir2* Regulates Lipid Droplet Proteome Associated Genes in a Gender-specific Manner

**Figure 3:**
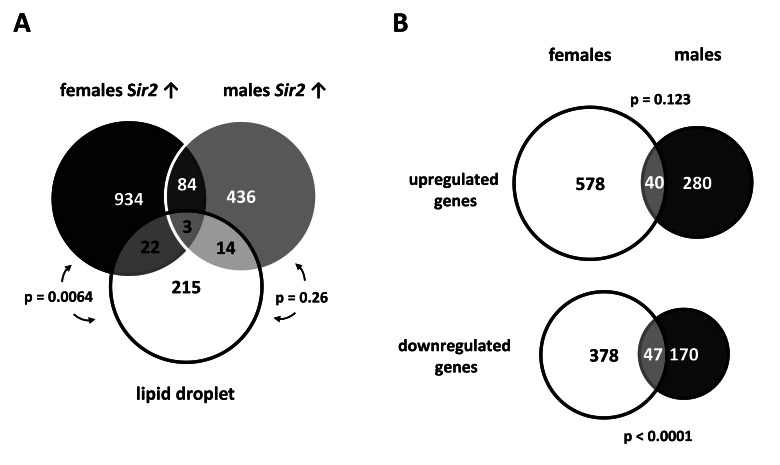
Gene expression profiles of fat bodies overexpressing *Sir2* show notable intersections with the lipid droplet proteome (**A**) Differentially regulated genes (up-and down-regulated) found in *Sir2* overexpressing males and females were compared with the lipid droplet proteome as defined by Beller et al. [25]. The Venn diagram shows considerable intersections between these different sample types (statistical analyses were performed with Fisher's exact test; see Table 2 for a list of intersecting genes). (**B**) To view gender-specific effects, the transcriptional profiles of fat bodies from UAS-*Sir2*>S_1_106-Gal4 males and females after 24 h of *Sir2* overexpression were compared. The scaled Venn diagrams depict the actual relationship of the numbers of up- and down-regulated genes of both samples and the number of overlapping genes. Genes which were up- or down-regulated > 1.5 fold in at least 2 of 3 microarrays were included in the diagrams shown in (**A**) and (**B**).

Based on the transcriptome studies performed with isolated fat tissue from *Sir2* overexpressing males and females, we focused on those genes that are relevant for fat tissues. Within this tissue lipid droplets are of central importance to regulate fat utilization [24]. Thus, we hypothesized that fat body *Sir2* modulates the composition of the lipid droplet proteome and compared the gene expression data from fat bodies overexpressing *Sir2* with a list of Gene IDs corresponding to the lipid droplet proteome as defined by Beller et al. [25]. Corresponding genes of 39 out of 254 lipid droplet associated proteins were found to be differentially regulated in fat bodies overexpressing *Sir2* which corresponds to 15 % of the lipid droplet proteome (Fig. 3A; p value 0.007). The (putative) functions of these genes are very diverse and range from lipid storage and fatty acid metabolism, stress response and energy metabolism, to chromatin assembly (Table 2).

**Table 2:** Gene IDs of proteins found to be associated with lipid droplets and genes found to be differentially regulated after *Sir2* overexpression in the fat body

Gene ID	Name	(Putative) function	Gender-specific regulation	
CG31618	His2A:CG31618	chromatin assembly or disassembly	♂ ↑	
CG5170	Dodeca-satellite-binding protein 1	chromosome segregation	♀ ↑	
CG2512	alpha-Tubulin at 84D	cytokinesis	♀ ↑	
CG18000	short wing	cytoplasmic dynein intermediate chain	♀ ↑	
CG1049	CTP:phosphocholine cytidylyltransferase 1	endocytosis	♀ ↓	
CG5915	Rab7	endosome to lysosome transport	♂ ↑	
CG11642	TRAM	phagocytosis	♀ ↑	
CG6543	CG6543	fatty acid β-oxidation	♀ ↑	
CG12262	CG12262	fatty acid β-oxidation	♀ ↓	
CG3523	Fatty acid synthase	fatty acid synthesis	♀ ↑	
CG1112	alpha-Esterase-7	lipid storage	♂ ↓	
CG5119	polyA-binding protein	mRNA 3'-UTR binding	♀ ↑	
CG9748	belle	ATP-dependent RNA helicase activity	♀ ↑	
CG9325	hu li tai shao	axon guidance	♀ ↓	
CG7920	CG7920	neurogenesis	♂ ↓	
CG3725	Calcium ATPase at 60A	neuromuscular synaptic transmission	♂ ↑	
CG8322	ATP citrate lyase	citrate metabolism	♀ ↑	
CG4233	Glutamate oxaloacetate transaminase 2	L-aspartate:2-oxoglutarate aminotransferase activity	♀ ↑	♂↑
CG7998	Malate dehydrogenase 2	TCA cycle	♀ ↑	
CG2968	lethal (1) G0230	hydrogen-exporting ATPase activity	♀ ↑	
CG1683	Adenine nucleotide translocase 2	mitochondrial ATP:ADP antiporter activity	♀ ↑	♂↓
CG7834	CG7834	oxidative phosphorylation	♂ ↑	
CG10639	CG10639	oxidoreductase activity	♀ ↑	
CG5590	CG5590	oxidoreductase activity	♀ ↑	
CG5809	calcium-binding protein 1	cell redox homeostasis	♂ ↑	
CG6988	Protein disulfide isomerase	protein disulfide isomerase activity	♀ ↑	
CG5834	Hsp70Bbb	stress response	♂ ↑	
CG5436	Heat shock protein 68	stress response	♂ ↓	
CG8937	Heat shock protein cognate 1	unfolded protein response	♀ ↑	
CG7808	Ribosomal protein S8	structural constituent of ribosomes	♀ ↑	
CG1524	Ribosomal protein S14a	structural constituent of ribosomes	♂ ↑	
CG4087	Ribosomal protein LP1	structural constituent of ribosomes	♂ ↑	
CG7014	Ribosomal protein S5b	structural constituent of ribosomes	♂ ↑	
CG9282	Ribosomal protein L24	structural constituent of ribosomes	♂ ↑	
CG3373	Hemomucin	biosynthetic process	♀ ↓	
CG6415	CG6415	glycine catabolism	♀ ↑	
CG2852	CG2852	reproductive process	♀ ↓	
CG18212	aluminum tubes	unknown	♀ ↑	♂↓
CG9186	CG9186	unknown	♂ ↓	

Gene IDs of proteins which are associated with lipid droplets [25] and of genes which were differentially regulated in 2 of 3 microarrays of fat bodies of female or male UAS-*Sir2*>S_1_106-Gal4 flies after feeding of RU486.

When comparing gene expression patterns of male and female fat bodies overexpressing *Sir2* we found great differences between genders. Only 40 of 619 genes up-regulated in females were also found within the 320 genes up-regulated in males (Fig. 3B, p value 0.123). This overlap corresponds to 6.5 % and 12.5 % of up-regulated genes in females and males, respectively. The situation was different for down-regulated genes where 11 % and 22 % were overlapping between females and males, respectively (p value < 0.0001). These gender-specific differences are mirrored in the comparison of genes affected by *Sir2* overexpression and the genes corresponding to proteins associated with lipid droplets. Considering the data for males and females separately 17 (7 %) and 25 (10 %) of the lipid droplet proteome genes are regulated, respectively (Fig. 3A). But only 1 gene (*Glutamate oxaloacetate transaminase 2*) is up-regulated in both (Table 2) whereas all other genes are not or inversely regulated in the respective other gender. Taken together, these results point to a role for fat body *Sir2* in modulating the composition of the lipid droplet proteome, which is obviously gender-specific.

### Fat Body *Sir2* Increases Starvation Resistance in Male Flies

**Figure 4:**
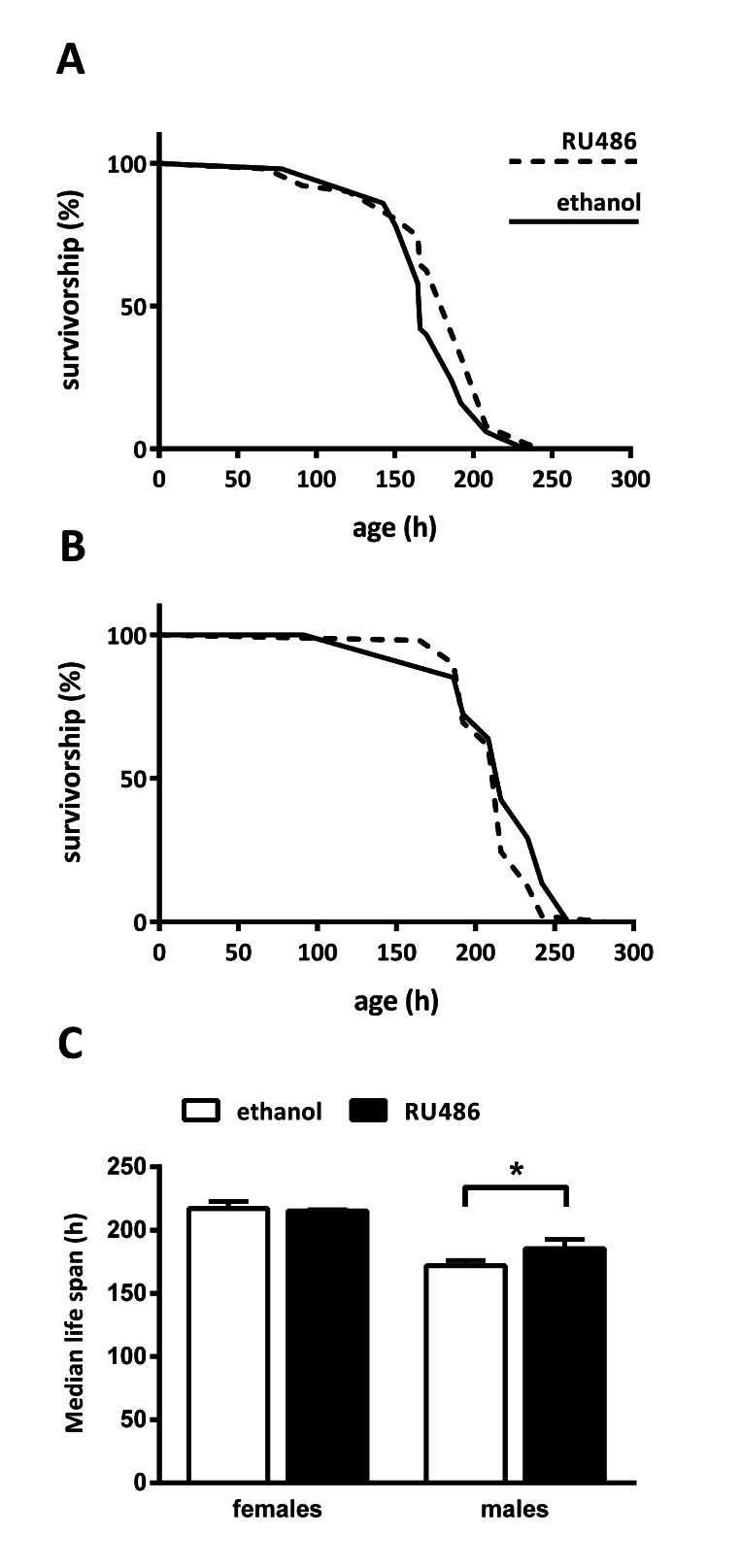
Fat body *Sir2* increases starvation survival in males Mated female (**A**) or male (**B**) UAS-*Sir2*>S_1_106-Gal4 flies fed on 1 % agar containing 400 μM RU486 or diluent (ethanol) and dead flies were scored 1-3 times a day. (**C**) Median lifespan of females was 217 ± 6 h and 215 ± 1 h in control and experimental flies, respectively. For males median lifespans were 172 ± 4 h and 185 ± 7 h for control and *Sir2* overexpressing flies, respectively. Data are represented as means + SEM. Log rank analysis was performed using GraphPad Prism 6.00, n = 50.

The fat body is the major storage organ of the fly, which regulates the response to differing nutrient conditions. *Sir2* is capable of sensing cellular energy levels, as its enzymatic activity is NAD^+^- dependent [26]. We hypothesized that fat body *Sir2* participates in regulating the response to changes in nutrient conditions when promoting longevity. One measure for the ability to react to changing nutrient supply is to determine starvation resistance. Mated UAS-*Sir2*>S_1_106-Gal4 flies fed on 1 % agar with or without RU486 and starvation medium was changed every 2-3 days. Dead flies were counted 1-3 times a day. Fat body *Sir2* appeared to have no impact on female starvation resistance as median lifespan of control and *Sir2* overexpressing flies showed no difference (Fig. 4A, C). However, fat body *Sir2* increased starvation survival in males by 13 % (Fig. 4B, C). In line with the transcript-tome analysis we found a small gender-specific reaction to starvation after *Sir2* overexpression in the fat body.

### No Influence of *Sir2* on Fat Storage and Fat Cell Morphology

As *Sir2* overexpression in the fat body did not only extend lifespan of the flies but also influenced the relative abundance of lipid droplet proteome associated genes and starvation resistance in a gender-specific manner we wanted to elucidate the role of fat body *Sir2* on fat storage and morphology of the fat cells. We tested if *Sir2* overexpression affects relative body fat content, body weight, the morphology of the fat cells and the size of the fat droplets in males and females.

**Figure 5:**
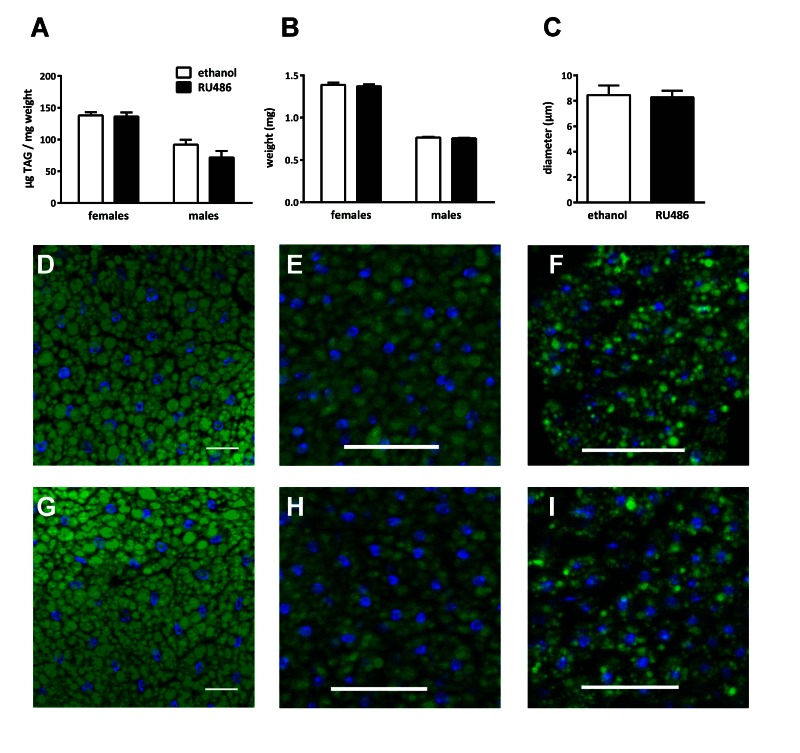
*Sir2* overexpression in the fat body did not affect fat storage and fat cell morphology Relative body fat content as quantified by coupled colorimetric assay (**A**) and body weight (**B**) of male and female UAS-*Sir2*>S_1_106-Gal4 flies feeding on medium with or without 400 μM RU486 showed no significant differences. Diameters of fat droplets were measured in fat bodies of UAS-*Sir2*>S_1_106-Gal4 L3 larvae after Bodipy staining (**C**) and no differences were observed. Bodipy staining furthermore reveals no differences in size and structure of fat cells as well as droplet size in L3 larvae (**D, G**), adult females (**E, H**) or males (**F, I**) after feeding on ethanol (top row) or RU486 (bottom row). **A-C**, Data are represented as means + SEM. Mann-Whitney-U-Test was performed using GraphPad Prism 6.00, n = 4 for coupled colorimetric assay and body weight, n = 7 for fat droplet diameter measurements. **D-I**, scale bar represents 50 μm.

Mated UAS-*Sir2*>S_1_106-Gal4 males or females were fed on medium containing RU486 or ethanol for 7 d before they were weighed and relative body fat content was quantified by a coupled colorimetric assay according to Hildebrandt et al. [27]. Neither sex showed fat body *Sir2* - dependent effects on fat storage (Fig. 5A) and body weight (Fig. 5B). To analyze changes in the morphology of the fat body we performed Bodipy staining with L3 larvae (Fig. 5 D, G), adult females (Fig. 5 E, H) and males (Fig. 5 F, I). Although fat cell size and filling levels of fat stores obviously varied substantially between all sample types, no effect of *Sir2* overexpression (Fig. 5 D-I, bottom row) was observed on these parameters compared to controls (Fig. 4 D-I, top row). We randomly selected 14 pictures comprising 7 biological replicates and measured diameters of fat droplets of the larval samples (Fig. 5C) to confirm the results of the qualitative analysis of the tissues. Altogether, these results do not reveal an obvious phenotype in fat body cells following *Sir2* over-expression, regardless of developmental stage or sex.

## DISCUSSION

The aim of this study was to test whether tissue-specific overexpression of *Sir2* in the fat body of adult flies can elongate lifespan and to elucidate the mechanisms by which fat body *Sir2* promotes longevity. We showed that moderate (roughly 3-fold) *Sir2* overexpression in the fat body can extend median lifespan of both male and female *Drosophila melanogaster* by about 13 %. With this study, we provide additional independent evidence for the concept of *Sir2* as a longevity gene in *Drosophila* as suggested by earlier studies [3, 4, 16, 17]. The UAS-line used in our study has not been employed for lifespan experiments so far and does not show increased expression of *dnaJ-H*, which has been shown to affect apoptosis control of *Sir2* and may also interfere with *Sir2* functions with regards to longevity [28]. Importantly, we used the inducible GeneSwitch Gal4 System; this removes the use of different genetic backgrounds in control and *Sir2* overexpressing flies, a potentially severe confounder in lifespan studies. Geneswitch also allows overexpression during adulthood only, thus removing any developmental testing potential gerontogenes from a therapeutic perspective. However, our results contradict the study from Burnett et al. [19] who did not observe an effect of ubiquitous overexpression of *Sir2* on *Drosophila* lifespan. This may be due to antagonistic effects on lifespan caused by Sir2 overexpression during development, and/or due to the site of overexpression; by restricting overexpression of *Sir2* to the fat body, possibly harmful effects caused by overexpression in other tissues may be excluded. In this context it is notable that overexpression of *Sir2* in the nervous system prolonged lifespan of flies [3]. These results are in line with the observation that only these two tissues express *SIR2* protein at high levels [3].

We also aimed to test different hypotheses of how fat body *Sir2* may confer longevity. Our first working hypothesis was to presume that *Sir2* overexpression mimics dietary restriction, which has been proposed by other studies [3, 17, 29]. This led us to compare microarray data generated from fat bodies overexpressing *Sir2* with those obtained from fat bodies of animals subjected to DR. As the induced transcriptional changes of both interventions were different our data did not confirm this hypothesis. Instead they support the alternative hypothesis that DR and *Sir2* overexpression are indendent processes. Furthermore, dietary restriction had no effect on *Sir2* mRNA levels in fat tissue. Our results join a canon of studies presenting conflicting results regarding this topic. Previous findings in yeast [30-33], flies [3, 17, 19] and rodents [34, 35] are inconsistent, which may be due to application of different dietary /caloric restriction regimes, different sites of ectopic expression or knockout of *Sir2* and/or genetic backgrounds. We used the food preparations recommended by Ja et al. [23] to circumvent adverse side effects caused by desiccation of the flies, which robustly produced a DR response in both sexes and the food used in the *Sir2* experiments was identical except for addition of RU486 dissolved in ethanol or ethanol alone. However, the data presented here suggest that fat body *Sir2* does not mediate the effects of DR on lifespan of *Drosophila*.

It has also been proposed that *Sir2* in the fat body plays a role in regulating fat storage and mobilization as *Sir2 / Sirt1* has been implicated in regulation of fat metabolism in flies and mammals. Firstly, we compared the part of the fat body's transcriptome that is altered by *Sir2* overexpression with those genes coding for the lipid droplet proteome as these organelles are known to be central players for the control of fat storage and mobilization [36-39]. A considerable overlap between these data sets suggested a role for fat body *Sir2* in modulating the composition of the lipid droplet proteome, a hypothesis that is supported by the statistical data. Nevertheless, as lipid droplets have been shown to regulate aging processes [40] our data provide a good starting point to unravel the putative role of *Sir2* in lipid droplet biology. The overlap with genes coding for lipid droplet proteins was highly sex specific, a feature that was also seen for starvation resistance and the *Sir2* induced transcriptional changes in fat tissues.

Our findings that fat storage, body weight and lipid droplet size were not changed after *Sir2* overexpression, contrast with other recent studies which demonstrated an impact of RNAi-mediated knockout of *Sir2* in the fat body or constitutive whole body knockout, respectively, on fat metabolism [16]. Furthermore, data from mouse studies indicate a role for *Sir2* in the regulation of lipid metabolism [8, 10]. These divergences may result from the different expression system used in our study. We aimed to overexpress *Sir2* only moderately in the fat body and nowhere else, and only during adulthood. This strategy should have only minimal confounding effects on e.g. fat storage caused by complete silencing of *Sir2* in either the fat body/liver or the whole animal.

Overall, we conclusively showed that *Sir2* is a longevity gene in *Drosophila*. With this study we provide additional independent evidence for its ability to extend lifespan of male and female flies when moderately overexpressed in the adult fat body. We also obtained transcriptional profiles elicited by this overexpression and propose a role for *Sir2* in lipid droplet biology especially under conditions of starvation. However, the precise conditions under which *Sir2* affects fat utilization and the molecular mechanisms involved in this function remain to be elucidated. Furthermore, our transcriptional data do not support the idea of *Sir2* mediating the response to dietary restriction. This study again highlights the relevance of *Sir2* for aging processes and its potential as a pharmacological target to cure age-related diseases.

## METHODS

### Fly Strains and Husbandry

Males of the driver line S_1_106-Gal4 (gift from R. Kuehnlein, MPI Goettingen, Germany [20]) were crossed to virgin females of UAS-*Sir2* (gift from Kyung-Tai Min,[28]) to generate flies overexpressing *Sir2* in the fat body after induction by RU486. As a control for RU486-dependent effects on lifespan a control crossing using the driver line and the *w^1118^* strain (Bloomington Drosophila Stock Center, # 5905) was performed. Fly stocks were raised on standard cornmeal-agar medium at 25 °C under a 12 h/12 h light/dark cycle.

### Lifespan Experiments

For lifespan experiments the F1 generation of the crossings mentioned above were allowed to mate for two days after eclosion before they were set on medium containing 5 % (w/v) yeast extract (Becton Dickinson), 5 % (w/v) sucrose, 8.6 % cornmeal, 0.5 % (w/v) agar, 0.03 % (v/v) propionic acid, 0.3 % (v/v) methyl-4-hydroxybenzoat and either 400 μM RU486 (diluted from a 10 mM stock in 90 % ethanol) for the experimental or the corresponding volume of 90 % ethanol for the control groups. Flies were kept at a density of 25 animals per vial (68 ml, Greiner Bio One) at 25 °C, 40-60 % humidity and a 12 h/12h light/dark cycle. Medium was changed every 3 – 4 days and dead flies were counted.

### Dietary Restriction (DR)

The DR experiments were performed with medium prepared as recommended in Ja et al. to provide sufficient water supply [23]. Control medium contained 5 % (w/v) yeast extract (Becton Dickinson), 5 % (w/v) sucrose, 8.6 % cornmeal, 0.5 % (w/v) agar, 0.03 % (v/v) propionic acid, 0.3 % (v/v) methyl-4-hydroxybenzoat. DR medium contained only 0.25 % yeast extract. *w^1118^* flies were allowed to mate for 2 days before genders were separated and either used for lifespan studies as described above or fed on control or DR medium for 7 d prior to dissection of fat bodies and RNA isolation.

### Microarray Analysis

Microarray analyses were essentially performed as described earlier [41, 42]. In brief, cDNA synthesis from RNA isolated from fat bodies was performed with Prime Script RT (Takara) using the following primers: OdT T7 I (5'-GAG AGA GGA TCC AAG TAC TAA TAC GAC TCA CTA TAG GGA GAT TTT TTT TTT TTT TTT TTT TTT T G/A/C-3') and CapFinder Sp6rG (5'-CAG CGG CCG CAG ATT TAG GTG ACA CTA TAG A rGrGrG-3'). cDNA was amplified with OdT T7 II (5'-GAG AGA GGA TCC AAG TAC TAA TAC GAC TCA CTA TAG G-3') and Adaptor Sp6rG (5'-GAC GCC TGC AGG CGA TGA ATT TAG G-3') and LA Taq polymerase [43]. cDNA was transcribed with MEGAscript® T7 (Ambion) including aminoallyl-UTP (Ambion) and subsequently labeled with Alexa Fluor 555 or 647 (Invitrogen) for control or experimental sample, respectively. Samples were hybridized to *Drosophila* 14 k V2 (Canadian *Drosophila* Microarray Centre, Toronto, Canada) and scanned in a GenePix 4000B Microarray Scanner (Axon Instruments, Molecular Devices). Data acquisition, normalization and analysis including hierarchical clustering were carried out with the corresponding programs GenePix 6.0 and Acuity 4.1 (Axon Instruments, Molecular Devices). Scaled Venn diagrams were drawn with Wolfram Mathematica [44]. The microarray data have been deposited at the GEO database (Gene Expression Omnibus (http://www.ncbi.nlm.nih.gov/geo/)) with the accession number GSE45974.

### Quantitative real-time PCR

Mated *Sir2*>S_1_106-Gal4 flies fed on medium containing RU486 or ethanol as described for lifespan experiments for 24 h. RNA was isolated from the abdomen of 3 animals for one biological replicate and reverse transcription was performed using OdT-T7I Primer and SuperScript III Reverse Transcriptase (Invitrogen). qPCR was performed in a StepOne Real-Time PCR System (Applied Biosystems) using the DyNAmo Flash SYBR Green qPCR Kit (Finnzymes) and the following primers: *Sir2* forward (5'-GCC TCC AGG ACA GTT AGC AG-3'), *Sir2* reverse (5'- CGT GAT ACC GGG AAT TGA AG-3'), *RpL32* forward (5'- TTG GCT TCG GTT TCC GGC AAG-3'), *RpL32* reverse (5'-ATC GAT CCG ACT GGT GGC GGAT-3'). *RpL32* was used as a reference gene and expression data were analyzed using the method described by Pfaffl [45].

### Relative Body Fat Quantification and Body Weight Measurement

A coupled colorimetric assay was performed using the Triglycerides Kit from Thermo Fisher Scientific (# 981786) according to the protocol from Hildebrandt et al. [27]. Briefly, after mating and feeding on medium containing RU486 or ethanol for 7 d, for one biological replicate 8 males or 5 females were weighed on a digital accuracy balance and homogenized in 1 ml 0.05 % Tween-20 with 3 zirconium beads (2,8 mm) for 2 min at 3.25 m/s in a Bead Ruptor 24 (Omni International). Lysates were heat-inactivated for 5 min at 70 °C, centrifuged and supernatant was incubated with the Triglyceride Solution for 30 min at 37 °C. Absorbance was measured at 562 nm and glyceryl trioleate served as a standard.

### BODIPY Staining and Determination of Diameters of Lipid Droplets

After feeding on RU486 or control medium for 24 h L3 larvae or adults generated by crossing UAS-*Sir2* flies to the driver line S1106-Gal4 were dissected, fixed and fat bodies were stained with a solution containing 1 μg/ml BODIPY (Invitrogen) and 5 μg/ml Hoechst (Sigma) for 30 min at room temperature. Slides were mounted in Mowiol/DABCO (Roth) and photographed with a Zeiss Axio imager 7.1 microscope equipped with a Zeiss Axiocam MRM camera within the next two days. 14 photographs taken from 7 biological replicates were analyzed per group with AxioVision 4.1 (Zeiss). To measure fat droplet diameters a line was drawn across each picture and only droplets intersecting with the line were measured using a measurement tool implemented in the program.

### Starvation Resistance

Mated *Sir2*>S_1_106-Gal4 flies were kept on 1 % agar containing either 400 μM RU486 or ethanol and vials were changed every 2-3 days. Dead flies were scored 1-3 times a day.

### Statistics and Bioinformatics

All statistics were performed using GraphPad Prism version 6.00 for Windows, GraphPad Software, La Jolla California USA, www.graphpad.com. Lifespan and starvation survival data were analyzed by log rank test and data from qPCR, body fat quantification, body weight and diameters of lipid droplets by Mann-Whitney-U Test. Statistical analyses of Venn-diagram data were performed with Fisher's exact test.

## SUPPLEMENTAL DATA



## Abbreviations

DR: dietary restriction
ns: not significant
