# Supplementary material for: Overexpression of Sir2 in the adult fat body is sufficient to extend lifespan of male and female Drosophila
Source: Aging (Albany NY). 2013 Apr 29;5(4):315–27. doi: 10.18632/aging.100553 (PMC3651523; doi:10.18632/aging.100553)
Supplement: Supplementary file 1 [file aging-05-315-s001.pdf]

has a negative effect on organismal growth and induces a peculiar set of target genes. BMC Genomics. 2010; 11:265.

43. Schramm G, Bruchhaus I and Roeder T. A simple and reliable 5'-RACE approach. Nucleic Acids Res. 2000; 28:E96.

44. Chandler S, Weisstein, EW. 2012. The perfect Venn diagram. Wolfram Demonstration Project.

45. Pfaffl MW. A new mathematical model for relative quantification in real-time RT-PCR. Nucleic Acids Res. 2001; 29:e45.

SUPPLEMENTAL DATA

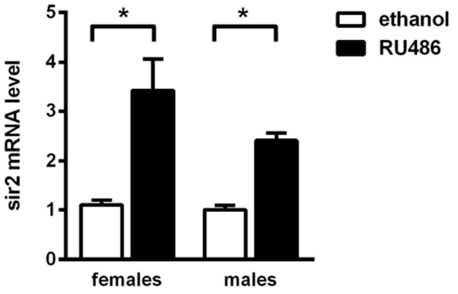

Figure S1. RU-486 induced weak overexpression of *Sir2* in the abdominal fat body of UAS-Sir2>S1106-Gal4 flies. Mated flies were fed on fly food containing ethanol or 400 µM RU486 for 24 h, fat bodies were dissected and qPCR was performed. Shown are means of 4 replicates + SEM. Mann-Whitney-U-Test was performed using GraphPad Prism 6.00. * p-value < 0.05.

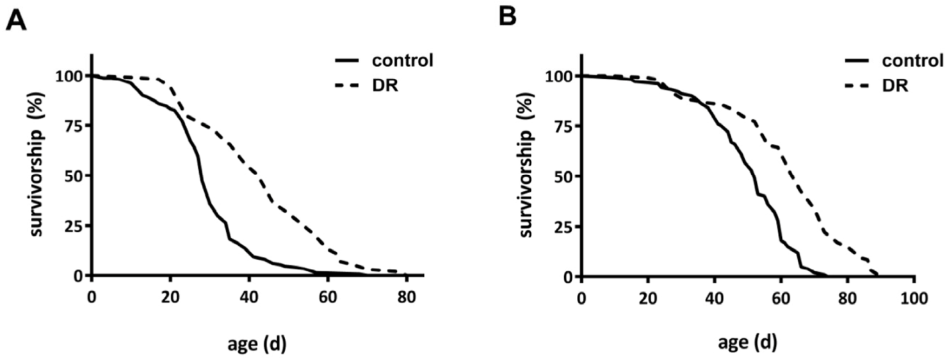

Figure S2. DR increased life span of Drosophila. Mated w1118 females (**A**) or males (**B**) fed on control medium (5 % yeast extract) or DR medium (0.25 % yeast extract) throughout adult life. n ≥ 95 for all groups. For results of log rank analysis see Table S2.

Table S2. DR increased life span *Drosophila*.

Food preparation	Gender	Median life span (d)	Median life span extension (% of control)	Maximum life span (d)	p-value	n
Control (5 % YE)	♀	28	-	63	-	98
DR (0.25 % YE)	♀	46	+ 64,3	77	<0,0001	99
Control (5 % YE)	♂	52	-	70	-	100
DR (0.25 % YE)	♂	66	+ 27,0	84	<0,0001	95

Data derived from log rank analysis of the survival curves shown in Fig. S2. The maximum life span is the median of 10 % survival. Log rank test was performed using GraphPad Prism 6.00. Experiments shown are representatives of two independent experiments. ns, not significant.
